# Environmental enrichment enhanced neurogenesis and behavioral recovery after stroke in aged rats

**DOI:** 10.18632/aging.205010

**Published:** 2023-09-08

**Authors:** Ji Yan, Yan Liu, Fangda Zheng, Dan Lv, Di Jin

**Affiliations:** 1 Department of Laboratory Medicine, The Fourth People’s Hospital of Shenyang of China Medical University, Shenyang, Liaoning, China; 2 Department of Neurology, The Fourth People’s Hospital of Shenyang of China Medical University, Shenyang, Liaoning, China; 3 Department of Laboratory Medicine, The Affiliated Hospital of Liaoning University of Traditional Chinese Medicine, Shenyang, Liaoning, China; 4 Department of Acupuncture (Neurology), The Affiliated Hospital of Liaoning University of Traditional Chinese Medicine, Shenyang, Liaoning, China

**Keywords:** aging, environmental enrichment, stroke, ET-1, neurogenesis, behavioral recovery

## Abstract

Background and Purpose: Age is identified as a significant prognostic factor for poorer outcome after stroke. However, environmental enrichment (EE) has been reported to promote functional recovery after ischemic stroke. The purpose of this study was to investigate whether environmental enrichment was beneficial to ischemic stroke in aged rats.

Methods: Aged rats were randomly assigned as control rats, rats subjected to cerebral ischemia, and rats with cerebral ischemia treated with EE for 30 days. Focal cortical ischemia was induced by intracranial injection of endothelin-1 (ET-1). EE housing began one day after focal ischemia and was maintained for the whole experimental period. We used immunofluorescence staining to analyze the neurogenesis in the subventricular zone (SVZ) and TdT-mediated dUTP-biotin nick-end labeling (TUNEL) assay to evaluate apoptosis. The expression of neuronal nuclei, glial fibrillary acidic protein (GFAP) and Iba-1 around the infarcted area were also measured by double immunohistochemistry.

Results: EE enhanced the proliferation of newborn neurons in the SVZ, as well as increased the long-term survival of newborn neurons. EE also exerted effects on inflammation after stroke. Furthermore, EE suppressed apoptosis and improved the motor functions after stroke in the aged rats.

Conclusions: EE improved post-stroke recovery on the basis of enhancing neurogenesis in aged rats.

## INTRODUCTION

Due to the increasing number of elderly people, stroke is a leading cause of death in recent years, which has brought a burden to society and economy [1]. Currently, a non-drug treatment has been used for the brain injury, primarily through interaction with the environment known as environmental enrichment (EE) [2–4].

EE promotes neuroplasticity through a combination of sensory, cognitive and motor stimuli, which is conducive to behavioral recovery after brain injury [5–7]. In EE, animals are housed in larger cages with a variety of new items that promote more physical activity, social interaction and exploration [8]. Over the past decade, impressive effects of EE have also been identified in other brain diseases such as Huntington’s disease (HD) [9], Alzheimer’s disease (AD) [10, 11] and various forms of brain injury. Besides, many studies have shown that EE has a positive effect on the stroke [12–14]. For instance, EE increased the proliferation of hippocampal and cortical endothelial cells [15], improved the survival rate of newborn astrocytes [16], decreased growth inhibitory molecules [17], induced neural plasticity [18] and promoted the formation of neovascularization in cerebral ischemia rats [19].

Altogether, the beneficial effects of EE after weaning and at adulthood have been well documented for ischemic stroke. However, age differences may affect neurogenesis, angiogenesis, inflammatory factors, and cerebral blood flow, thus affecting the accuracy of experimental results in ischemic animals. This may explain why drugs that work well in animal models do not show efficacy in stroke survivors [20]. Therefore, it is more appropriate to simulate ischemia model with old animals from the perspective of clinical transformation. In our study, we tested the hypothesis that EE can promote neurogenesis in older stroke rats, thereby promoting behavior recovery.

## MATERIALS AND METHODS

### Animals

Aged (18–20 months) male Sprague–Dawley rats (600–800 g) were used in the study. The rats were randomly assigned to three groups: sham-operated rats (*n* = 6, SHAM), rats subjected to cerebral ischemia (*n* = 8, ISC), and rats with cerebral ischemia treated with EE (*n* = 8, EE). Anesthesia was induced using a mixture of 3% isoflurane in 30% oxygen and 70% nitrous oxide and animals were maintained with 1.5% isoflurane for the surgeries [21, 22]. The study was reported according to the Animal Research: Reporting of *In Vivo* Experiments (ARRIVE) guidelines. All animal experiments were approved by the Use Committee and Animal Protection of China Medical University and carried out in accordance with the National Institutes of Health Guide for the Care.

### Endothelin-1 (ET-1) stroke model

The rat model of cerebral infarction was established by intracranial injection of ET-1. The vasoconstrictive peptide, ET-1 (Sigma, USA), was dissolved in sterile saline at a concentration of 0.5 μg/μL, and 4.5 μL was slowly injected (0.5 μl/min) at the following three stereotaxic coordinates: 1) AP +3.5 mm, ML +2.8 mm, DV −1.0 mm (1.5 μl); 2) AP +2 mm, ML +2.8 mm, DV −1.0 mm (1.5 μl); and 3) AP +0.5 mm, ML +2.8 mm, DV −1.0 mm (1.5 μl) [23]. The needle was left *in situ* for 3 min after injection before being slowly removed to avoid aspirating the ET-1 back through the needle tract [24]. The sham animals were injected with saline instead of ET-1.

### Environmental enrichment (EE)

The groups of sham-operated rats and ischemia rats were housed in standard cages (550 × 350 × 200 mm, 3 to 4 rats in each cage). For the EE group, aged rats were placed in a large cage equipped with a running wheel, catwalk, playing toys and hiding tunnel. The purpose was to offer the animals an opportunity to have complex sensorimotor stimulation and motor training. Enriched-environment housing began one day after ischemia.

### 5-Bromo-2-deoxyuridine (BrdU) labeling

**Figure 1:**
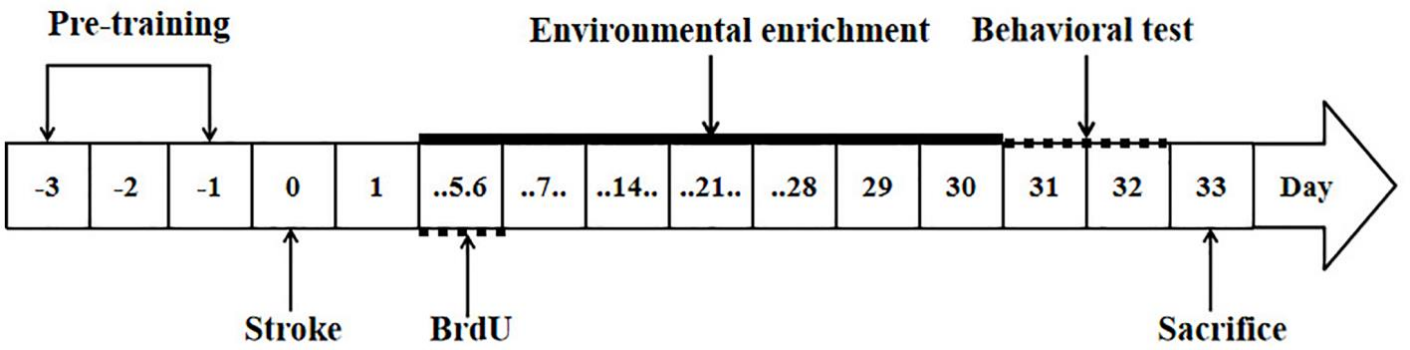
**Study design.** The arrows indicate the timing of pre-training, induction of stroke, BrdU labeling, environmental enrichment treatment, behavioral testing and sacrifice.

To label newly generated cells, all rats received two intraperitoneal injections of BrdU (100 mg/kg, Sigma–Aldrich, St. Louis, MO, USA) every day on postoperative days 5–6 [25] (Figure 1).

### Tapered/ledged beam-walking test

Changes in forelimb function were assessed using a tapered/ledged beam [26]. The rats were pre-trained for 3 days to traverse the beam before ischemia induction. Performance in the beam walking test was videotaped and later analyzed by calculating the slip ratio of the impaired (contralateral to lesion) forelimb (number of slips/number of total steps) [22] (postoperative day 31–32, Figure 1).

### Tissue preparation

After the 33 day follow-up, the rats were perfused through the heart, and then the brains were dissected and fixed. A series of consecutive 40 μm thick sections were cut from the anterior brain mass on a cryoknife (Thermo Electron, Waltham, MA, USA) for immunostaining.

### Measurement of infarct volume

Remove from the coronal sections (40 μm) at 1 mm intervals from +4.5 to 2.5 mm from the bregma. Sections were mounted on slides, air dried, and stained with cresol violet (Sigma, St. Louis, MO, USA) [27]. Contralateral and ipsilateral hemispheric areas were measured using NIH Image J. The complete area of the ipsilateral (damaged) hemisphere was subtracted from the contralateral hemisphere area of each section, and the area was multiplied by the distance between sections to obtain the total infarct volume.

### Immunohistochemistry

NeuN/BrdU, GFAP/BrdU and Iba-1/BrdU positive cells around the infarcted area, DCX/BrdU positive cells in the subventricular zone (SVZ) were observed by immunofluorescence staining. Immunofluorescence staining was performed by free floating method [22]. Immunofluorescence staining antibody concentrations were as follows: BrdU was detected using sheep anti-BrdU (1:500), anti-doublecortin (DCX) (1:800), mouse anti-neuronal nuclei (NeuN) (1:500), rabbit anti-Iba-1 (1:500) or rabbit anti-glial fibrillary acidic protein (GFAP) (1:1000). Cell apoptosis was detected by TUNEL method according to the instructions of Roche apoptosis Kit (*In situ* cell death detection kit; Roche).

### Image processing and analysis

Brain sections from +0.96 mm to −0.24 mm at the bregma level were taken from each rat, with an interval of 6. A total of 5 slices were taken from each rat. DCX/BrdU positive cell images were collected in the SVZ of the infarcted side with a confocal microscope (Leica SP2, FV-1000, Germany) at 20 times magnification, and counted with NIH Image J software. Brain sections from + 1.92 mm to 0.6 mm at the bregma level were taken from each rat, with an interval of 6. A total of 5 slices were taken from each rat. BrdU/NeuN, BrdU/GFAP and BrdU/Iba-1 positive cells were collected in the cortex around the infarct with a confocal microscope (Olympus FV-1000, Japan) at a magnification of 100 times and 40 times respectively, and counted with NIH Image J software. Brain sections from −3.0 mm to −3.96 mm at the level of bregma were taken from each rat, with an interval of 6. A total of 4 slices were taken from each rat. Brain sections from + 1.92 mm to 0.6 mm at the bregma level were taken from each rat, with an interval of 6. A total of 5 slices were taken from each rat. TUNEL positive cell images were collected in the cortex around the infarct with a microscope at a magnification of 20 times, and counted with NIH Image J software. The results were expressed as the mean per high power field (HPF).

### Statistics

SPSS version 25.0 was used for the statistical analysis. Data were presented as the mean ± SD. All data were analyzed using one-way ANOVA. Use the least significant difference (LSD) post hoc test to analyze statistical differences between groups. *P* < 0.05 was considered a statistically significant difference.

### Availability of data and materials

The original contributions proposed in the study are included in the articles/supplementary materials and can be further inquired from the corresponding authors.

## RESULTS

### Operation

Four rats (25%) died after ET-1 lesions, which were excluded from further evaluation. All survived rats show the symptoms of neurological impairment which were observed for circling behavior if pulled gently by the tail. All sham-operated rats survived. So there were 6 rats in each ISC and EE group.

### Infarct volumes measurement

Typical ET-1 induced ischemic infarction includes extensive cortical damage. There was no significant difference in cerebral infarction volume between ISC group (58.2 ± 6.2 mm^3^) and EE group (53.4 ± 7.2 mm^3^) (*P* > 0.05).

### Effect of EE on the proliferation and survival of new cells in the SVZ of the aged rats after cerebral ischemia

**Figure 2:**
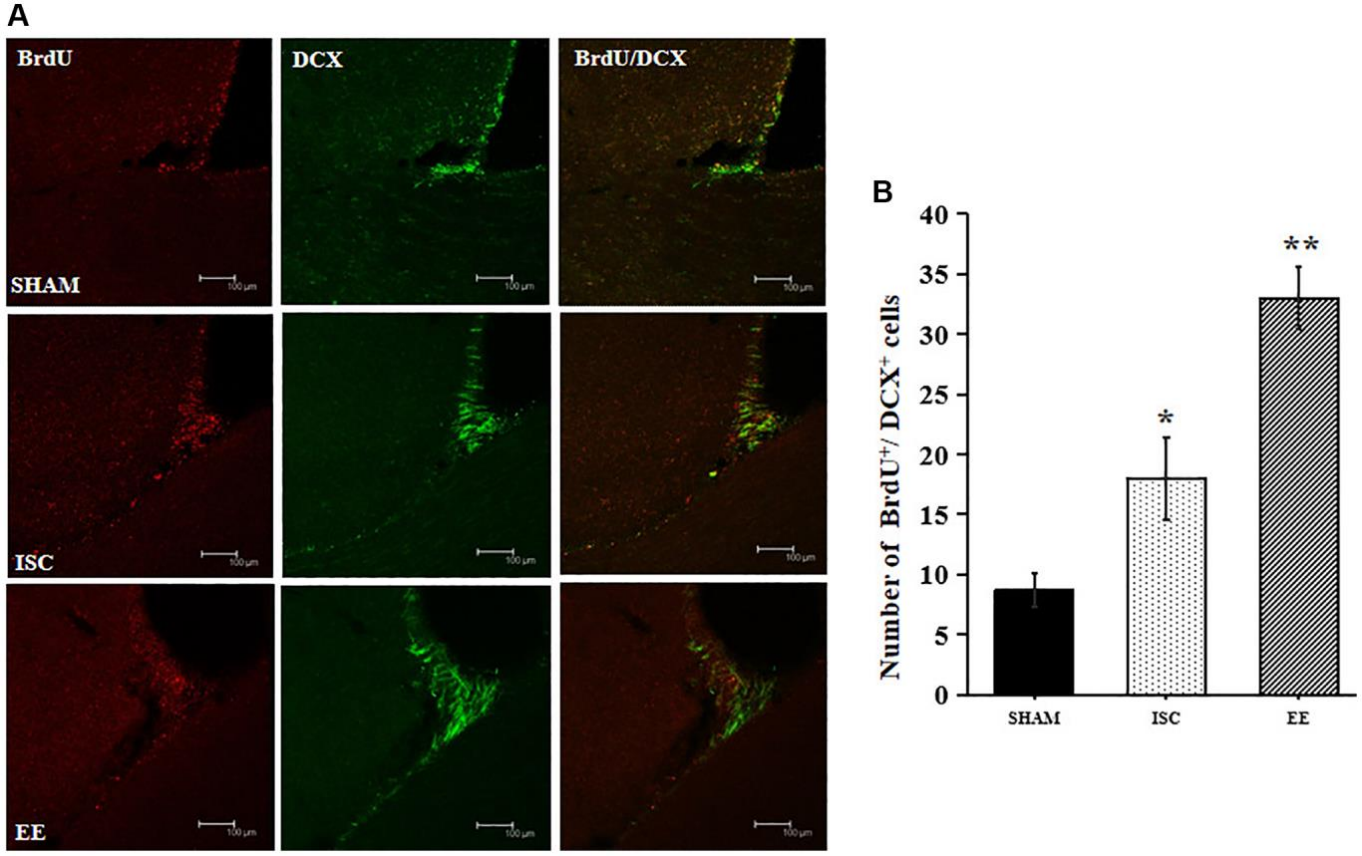
**Proliferation of newborn neurons in the SVZ.** (**A**) Representative confocal images for BrdU^+^/DCX^+^ cells in the SVZ. Scale bar = 100 μm. (**B**) Quantification of BrdU^+^/DCX^+^ cells in the SVZ (*n* = 6). Statistical significance: ^*^P < 0.01 vs. SHAM, ^**^P < 0.01 vs. ISC.

EE enhanced the proliferation of newborn neurons in the SVZ. DCX is a marker of neural precursor cells, and BrdU is a marker of cell proliferation. BrdU^+^/DCX^+^ double labeled cells represent neural precursor cells born after ischemia (Figure 2). There was a significant overall group effect in the number of the BrdU^+^/DCX^+^ cells in the SVZ (*F*_(2,15)_ = 131.27; *P* < 0.01). BrdU^+^/DCX^+^ cells in the SVZ were significantly increased in the ISC group compared with the SHAM group (18.0 ± 3.5 vs. 8.7 ± 1.4/HPF, *P* < 0.01). The number of BrdU^+^/DCX^+^ cells in EE group was higher than that in ISC group (33.0 ± 2.6/HPF, *P* < 0.01).

**Figure 3:**
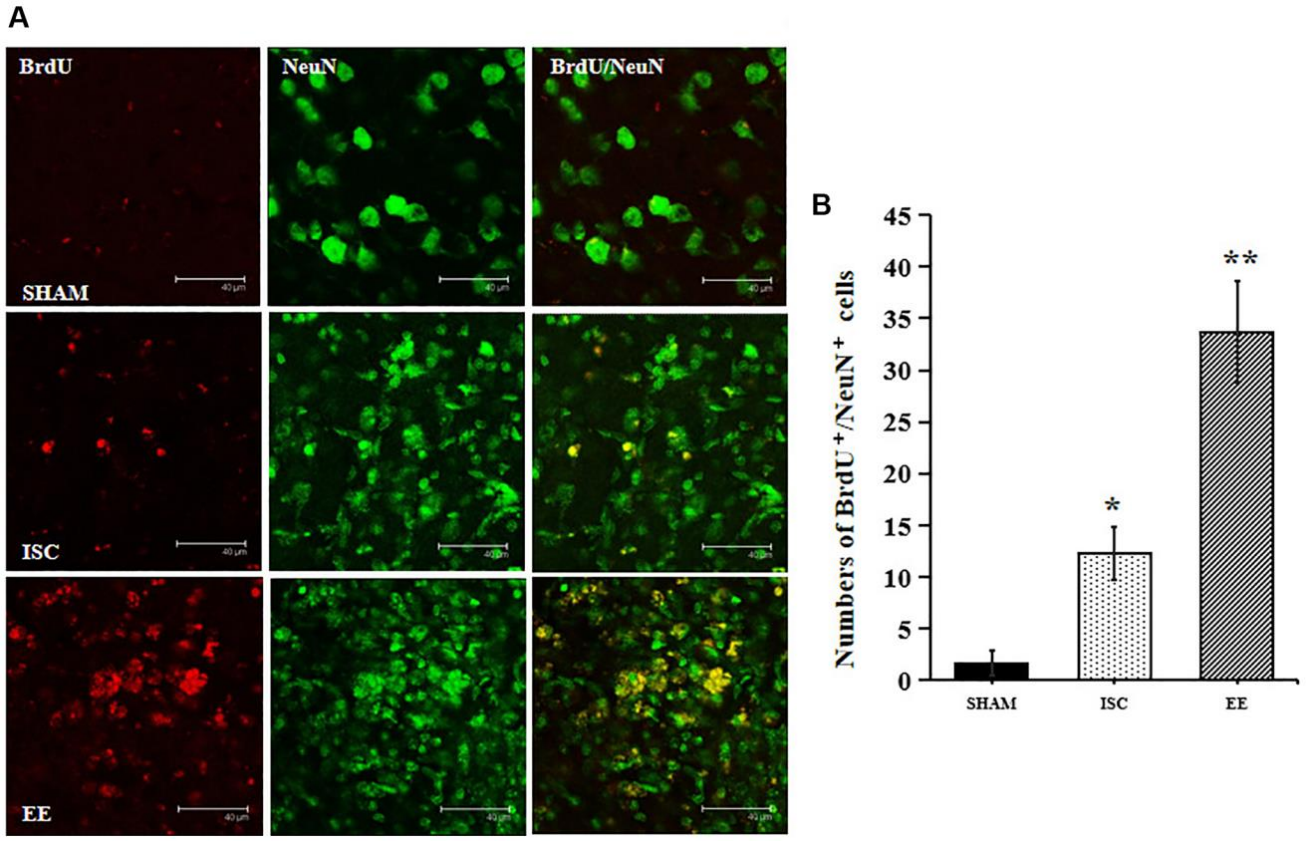
**The survival of neuroblasts in the peri-infarct cortex.** (**A**) Representative confocal images for BrdU^+^/NeuN^+^ cells in the peri-infarct cortex. Scale bar = 40 μm. (**B**) Quantification of BrdU^+^/NeuN^+^ cells in the peri-infarct cortex (*n* = 6). Statistical significance: ^*^P < 0.01 vs. SHAM, ^**^P < 0.01 vs. ISC.

EE enhanced the long-term survival of newborn neurons. NeuN is a marker of mature neural cells, and BrdU^+^/NeuN^+^ double labeled cells represent mature neural cells born after cerebral ischemia. The number of cells by BrdU^+^/NeuN^+^ double staining can be used to observe the survival of newborn neural cells (Figure 3). There was a significant overall group effect in the number of BrdU^+^/NeuN^+^ cells around the infarcted area (*F*_(2,15)_ = 149.33; *P* < 0.01). The number of BrdU^+^/NeuN^+^ cells around the infarcted area in the ISC group was significantly higher than that in the SHAM group (12.3 ± 2.6 vs. 1.7 ± 1.2/HPF, *P* < 0.01). EE further increased the number of BrdU^+^/NeuN^+^ cells (33.7 ± 4.9/HPF, *P* < 0.01) around the infarcted area after cerebral ischemia.

### Effect of EE on the proliferation of GFAP and Iba-1 in the infarcted area of the aged rats after cerebral ischemia

**Figure 4:**
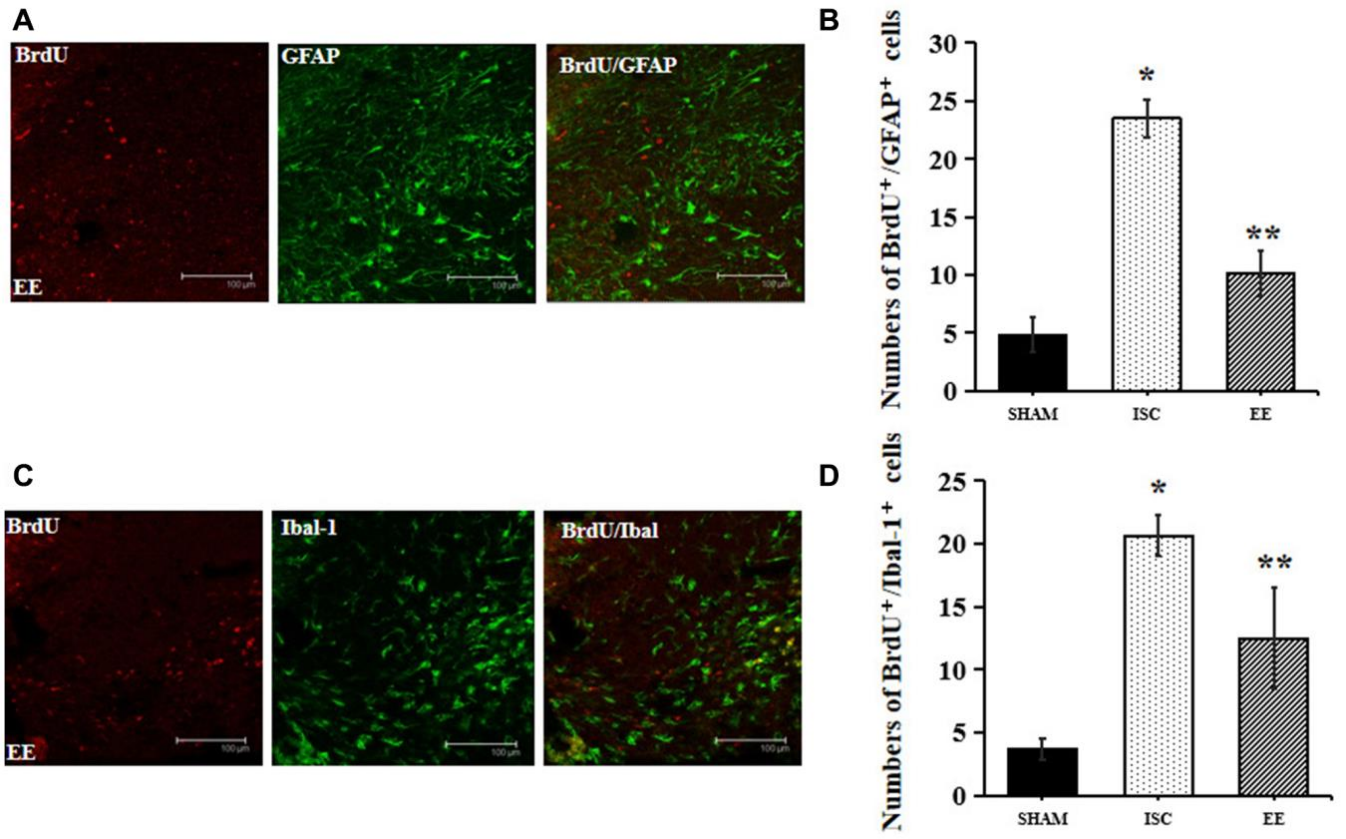
**Differentiation of neuroblasts in the peri-infarct cortex.** (**A**) Representative confocal images for BrdU^+^/GFAP^+^ cells in the peri-infarct cortex. Scale bar = 100 μm. (**B**) Quantification of BrdU^+^/GFAP^+^ cells in the peri-infarct cortex (*n* = 6). Statistical significance: ^*^P < 0.01 vs. SHAM, ^**^P < 0.01 vs. ISC. (**C**) Representative confocal images for BrdU^+^/Iba-1^+^ cells in the peri-infarct cortex. Scale bar = 100 μm. (**D**) Quantification of BrdU^+^/Iba-1^+^ cells in the peri-infarct cortex (*n* = 6). Statistical significance: ^*^P < 0.01 vs. SHAM, ^**^P < 0.01 vs. ISC.

GFAP is a marker of astrocytes and Iba-1 is a marker of microglia. The expression of Iba-1 and GFAP can be used to observe the effect of EE on microglia and astrocytes after cerebral ischemia (Figure 4). EE also exerted effects on inflammation after stroke.

There was a significant overall group effect in the number of BrdU^+^/GFAP^+^ cells around the infarcted area (*F*_(2,15)_ = 192.74; *P* < 0.01). The number of BrdU^+^/GFAP^+^ cells around the infarcted area in the ISC group was significantly higher than that in the SHAM group (23.5 ± 1.6 vs. 4.8 ± 1.5/HPF, *P* < 0.01). EE decreased the number of BrdU^+^/GFAP^+^ cells (10.2 ± 1.9/HPF, *P* < 0.01) around the infarcted area after cerebral ischemia.

There was a significant overall group effect in the number of BrdU^+^/Iba-1^+^ cells around the infarcted area (*F*_(2,15)_ = 66.27; *P* < 0.01). The number of BrdU^+^/Iba-1^+^ cells around the infarcted area in the ISC group was significantly higher than that in the SHAM group (20.7 ± 1.6 vs. 3.7 ± 0.8/HPF, *P* < 0.01). EE decreased the number of BrdU^+^/Iba-1^+^ cells (12.5 ± 4.0/HPF, *P* < 0.01) around the infarcted area after cerebral ischemia.

### EE can significantly inhibit apoptosis of infarcted area in aged rats after cerebral ischemia

**Figure 5:**
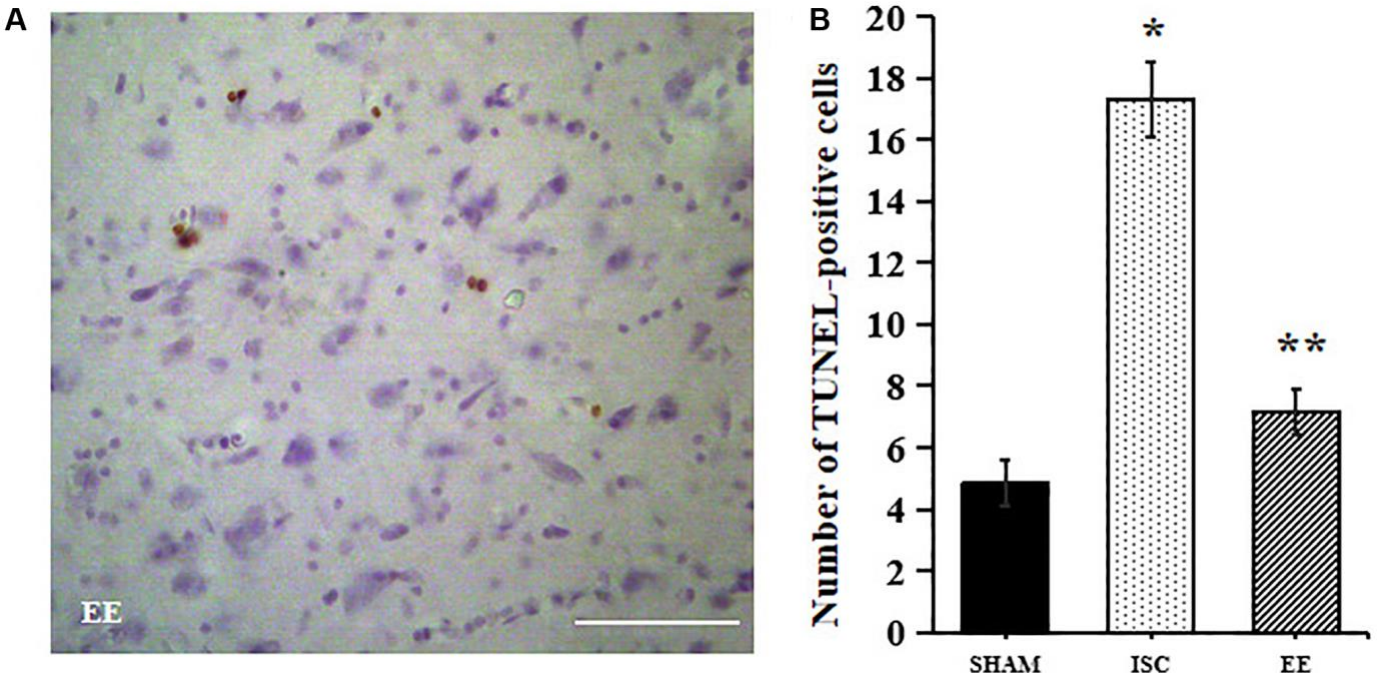
**Apoptosis in the peri-infarct cortex.** A Representative images for TUNEL-positive cells in the peri-infarct cortex. (**A**) Representative images for TUNEL-positive cells in the peri-infarct cortex. Scale bar = 50 μm. (**B**) Quantification of TUNEL-positive cells in the peri-infarct cortex (*n* = 6). Statistical significance: ^*^P < 0.01 vs. SHAM, ^**^P < 0.01 vs. ISC.

TUNEL method was used to detect apoptosis around the infarcted area (Figure 5). There was a significant overall group effect in number of the TUNEL-positive cells around the infarcted area (*F*_(2,15)_ = 305.8; *P* < 0.01). The number of TUNEL positive cells in ISC group was higher than that in SHAM group (17.3 ± 1.2 vs. 4.8 ± 0.8/HPF, *P* < 0.01). After 4 weeks of EE, TUNEL positive cells decreased (7.2 ± 0.8/HPF, *P* < 0.01).

### EE can promote the recovery of motor function in aged rats after cerebral ischemia

**Figure 6:**
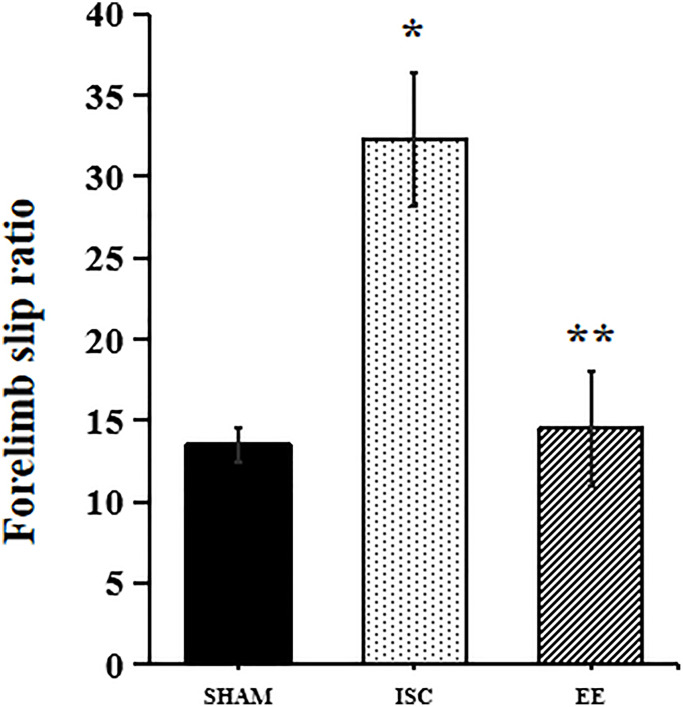
**Performance in behavioral tests.** Forelimb slip ratio in the beam-walking (*n* = 6). Statistical significance: ^*^P < 0.01 vs. SHAM, ^**^P < 0.05 vs. ISC.

We also used the beam walking test to detect the recovery of motor function in aged rats (Figure 6). In the beam-walking test, there was a significant overall group effect in slip ratio with the impaired forelimb (*F*_(2,15)_ = 67.22; *P* < 0.05). The beam walking test showed that ischemia could cause significant motor function damage in the aged rats in the ISC group (32.3 ± 4.1% vs. 13.5 ± 1.1%, *P* < 0.01). However, EE could reduce the error rate of the forepaw passing through the balance beam in aged rats after cerebral ischemia (14.5 ± 3.5%, *P* < 0.05).

## DISCUSSION

In our present study, we found that after intracerebral injection of ET-1 induced cerebral infarction, neuroblast generation was increased in the SVZ region in aged rats. After stroke, EE promoted the production of more neuroblasts in SVZ and improved the survival of newborn neurons in the peri-infarct area while reducing apoptosis. Furthermore, post-stroke rats displayed more severe neuroinflammation than those treatment of EE in aged rats, implying that EE have anti-inflammation properties. More importantly, behavioral assessments showed that improved beam walking performance may be related to the proliferation of newborn nerve cells. Consistent with previous studies, EE did not reduce infarct volume in ischemic aged rats [28].

Recent study points that EE upregulated BDNF expression in the MCAO rats [29], which may contribute to increase dendrite branching [30], enhance synaptogenesis [31], and increase the number of synapses with perforated postsynaptic densities [32]. However, those studies showed that the positive effects of EE on dentate gyrus. To the best of my knowledge, this is the first study demonstrated that the number of newly generated nerve cells in the SVZ was increased in the aged rats after EE compared with ischemic rats. These results suggested that newborn neurons after stroke are directed to the site of brain injury and participated in brain repair and functional recovery. In addition, our study showed there were BrdU^+^/NeuN^+^cells in the peri-infarct area of the aged rats one month after infarct, accompanied by more TUNEL-positive cells. The apoptosis of aged brain is faster than that of adult brain after stroke, which confirms that the apoptosis of new neurons after stroke is related to the increase of age [33]. These results support the idea that implementing an enriched environment after stroke may induce a favorable microenvironment for the development of the ischemic brain in old age. These results supported the idea that EE after stroke may induce a favorable microenvironment for the development of the aged ischemic brain.

Astrocytes are the most abundant of all brain cells [34]. In astrocytes, the expression of GFAP and vimentin increases with age [35, 36]. Moreover, the astrocytes had obvious hypertrophic morphological changes, and the cell bodies and protrusions were enlarged. After cerebral ischemia, the astrocytes and activated microglia around the lesion form glial scars, preventing the formation of new axons and blood vessels in the infarct area [37]. Histologically, microglia and astrocytes were gradually activated in young rats, peaking at days 14 to 28 and forming glial scars, whereas in older rats they responded more quickly, peaking in the first week after stroke. However, in terms of behavior, stroke injury was more severe in aged rats than in younger rats, and functional recovery was weaker [38]. Oligodendrocytes are strongly activated during early stage post-infarct remodelling in all rats, but this activation persists in aged rats [39]. The higher inflammatory response in the acute phase of ischemic stroke in aged mice is related to more serious neuronal damage and long-term behavioral dysfunction [40]. Therefore, the abnormal development of glial scar accelerates in aged rats, which is consistent with the stagnation of recovery in these animals. These results suggest that the transient abnormal neuroglial reaction after cerebral ischemia may lead to premature formation of scar tissue and hinders the recovery of nerve function after cerebral ischemia in aged rats [41, 42]. However, the treatment of EE in aged rats can reduce the proliferation of microglia and astrocytes and promote behavioral rehabilitation, which is consistent with previous studies [43, 44].

The significance limitation of the study is that there is no discussion on mechanism. Recently, the MAPK pathway has been confirmed to be closely related to the inflammatory response in nervous system diseases. Studies have confirmed that EE preconditioning can have a protective effect on acute ischemic stroke by inhibiting p38 MAPK/STAT1 pathway [45]. In addition, EE promotes functional recovery after stroke by inhibiting calpain 1 activity [46]. Another result revealed that the NF-κB/IL-17A signaling pathway plays an important role in EE-mediated a favorable microenvironment after ischemic stroke [47]. The underlying molecular mechanisms would have to be determined in our future research. In addition, one limitation of this study is that we did not analyze fMRI imaging results in elderly ischemic rats, it will analyze the role of EE in the recovery of elderly stroke from a radiological perspective in terms of structural changes, making the research results more meaningful and convincing, and can be also used as a longitudinal research observation EE in the occurrence and development of acute stroke in elderly rats. Another limitation of this study is the small number of aged rats used in this study. In our future research, it is necessary to increase the sample size and the results are likely to be more reliable.

In conclusion, this study shows that EE improved post-stroke recovery on the basis of enhancing neurogenesis in aged rats. Overall, these results indicated that EE is a practical and effective method to improve post-stroke recovery in aged rats. This may clarify the relevant mechanisms of modulation of brain plasticity after stroke in the elderly at the cellular level, and further understand stroke rehabilitation from the perspective of transformation. In addition, there are a number of clinical trials with stroke survivors that provide initial support for implementing EE in terms of increased sensory, motor and cognitive function. In the future, large sample size clinical studies should be conducted to confirm that the efficiency of EE in promoting stroke rehabilitation in elderly patients.
